# Supplementary material for: Research on hydrodynamic performance of S-type turbine based on linear wave
Source: PLoS One. 2025 Jan 3;20(1):e0310478. doi: 10.1371/journal.pone.0310478 (PMC11698312; doi:10.1371/journal.pone.0310478)
Supplement: S1 Nomenclature — (DOCX) [file pone.0310478.s002.docx]

| **Nomenclature** | |  |  |
| --- | --- | --- | --- |
| $C_{p}$ | power coefficient（-） | $T$ | a torque（N-m） |
| $P_{\text{wave}}$ | the incident wave power（w） | $n$ | a rotating speed（rpm） |
| $P_{c}$ | the dissipation power（w） | h | wave height |
| $P_{r}$ | the reflected wave power（w） | t | time |
| $P_{t}$ | the transmitted wave power（w） |  |  |
| $P_{s}$ | the S-type turbine power（w） |  | **Greek Symbols** |
| $H_{i}$ | the incident wave height（m） | $\rho$ | the water density(kg/m^3^) |
| $C_{g}$ | the group velocity（m/s^2^） | $g$ | the gravitational acceleration（m/s^2^） |
| $T$ | the incident wave period（s） |  | **Abbreviation** |
| $m_{i}$ | the turbine mass（kg） | S-type turbine | Savonius type turbine |
| $m_{d}$ | the end disc mass（kg） | DFBI | Dynamic Fluid Body Interaction |
| *d* | the blade diameter（m） | CFD | Computational Fluid Dynamics |
| *e* | the overlap ratio（-） | STDEV | standard deviation |
| *D_d_* | the end disc diameter（m） | TSR | tip speed ratio |
| $W_{s}$ | the rotational energy of the S-type turbine（J） | Max | maximum |
